# Analysis of Down syndrome newborn outcomes in three neonatal intensive care units in Rio de Janeiro, Brazil

**DOI:** 10.1590/1806-9282.20231186

**Published:** 2024-04-22

**Authors:** Hanna Gabriela da Cruz Alfaro, Saint Clair Gomes, Renato Augusto Moreira de Sá, Edward Araujo

**Affiliations:** 1 Fernandes Figueira Institute, Oswaldo Cruz Foundation, Department of Obstetrics and Fetal Medicine – Rio de Janeiro (RJ), Brazil.; 2 Universidade Federal Fluminense, Department of Obstetrics – Niterói (RJ), Brazil.; 3 Universidade Federal de São Paulo, Paulista School of Medicine, Department of Obstetrics – São Paulo (SP), Brazil.; 4 Universidade Municipal de São Caetano do Sul, Discipline of Woman Health – São Paulo (SP), Brazil.

**Keywords:** Down syndrome, Newborn, Intensive care units, Congenital anomalies

## Abstract

**OBJECTIVE:**

The aim of this study was to analyze the outcomes of newborns with Down syndrome admitted to three neonatal intensive care units in the city of Rio de Janeiro, Brazil.

**METHODS:**

A retrospective cohort study was conducted by analyzing the medical records between 2014 and 2018 of newborns with Down syndrome admitted to three neonatal intensive care units. The following variables were analyzed: maternal and perinatal data, neonatal malformations, neonatal intensive care unit intercurrences, and outcomes.

**RESULTS:**

A total of 119 newborns with Down syndrome were recruited, and 112 were selected for analysis. The most common maternal age group was >35 years (72.07%), the most common type of delivery was cesarean section (83.93%), and the majority of cases were male (53.57%). The most common reasons for neonatal intensive care unit hospitalization were congenital heart disease (57.66%) and prematurity (23.21%). The most common form of feeding was a combination of human milk and formula (83.93%). The second most common malformation was duodenal atresia (9.82%). The most common complications during neonatal intensive care unit hospitalization were transient tachypnea of the newborn (63.39%), hypoglycemia (18.75%), pulmonary hypertension (7.14%), and sepsis (7.14%). The mean length of stay in the neonatal intensive care unit was 27 days. The most common outcome was discharge (82.14%). Furthermore, 12.50% of newborns were transferred to an external neonatal intensive care unit, and 6% died.

**CONCLUSION:**

Newborns with Down syndrome are more likely to be admitted to the neonatal intensive care unit, and the length of hospital stay is longer due to complications related to congenital malformations common to this syndrome and prematurity.

## INTRODUCTION

Occasionally, chromosomes can undergo some changes during the mitotic and meiotic processes, resulting in aneuploidies^1^. Down syndrome (DS) is a genetic disorder caused by an imbalance in the chromosomal constitution during the fetal stage. It was described in 1866 by John Longdon Down^1^, and its etiology is related to an excess of genetic material resulting from an extra chromosome in pair 21^2^. Its estimated prevalence is 1/700 births, occurring worldwide, regardless of social or ethnic class, and it is considered the most common chromosomal abnormality in newborns^3,4^.

Some of the complications associated with DS include generalized hypotonia, neuropsychomotor development, thyroid disorders, hematological disorders, hearing/vision disorders, and orthopedic disorders^5,6^. Gastrointestinal malformations such as duodenal atresia and esophageal atresia are very common^5^. However, the major congenital malformation associated with the clinical course of these newborns is congenital heart disease (CHD), which is present in approximately 40–50%^7^ of newborns with DS, most commonly a complete or incomplete atrioventricular septal defect (AVSD)^8^.

The presence of congenital anomalies associated with DS, mainly CHD, increases the risk of admission to the neonatal intensive care unit (NICU) after birth and may directly affect the length of stay and outcomes^9^. Risk factors independently associated with primary NICU admission included the antenatal diagnosis of DS, the presence of CHD, pulmonary hypertension (PH), and the need for ventilation^9^. According to Mann et al.^10^ neonates with DS are five times more likely to be admitted to the NICU than those born with a normal karyotype (46% vs. 9.2%).

The objective of this study was to evaluate the neonatal outcomes of DS in three NICUs in Rio de Janeiro, Brazil.

## METHODS

A retrospective cohort study was conducted, reviewing the medical records of newborns with DS in three NICUs in the city of Rio de Janeiro, Brazil, between January 2014 and December 2018. Inclusion criteria were newborns admitted to the NICU within the first 24 h of life. Exclusion criteria were medical records of newborns transferred to external units, twin pregnancies, incomplete medical records, and medical records that were not available at the time of data collection. This study was approved by the Ethics Committee of the National Institute of Women's Health Child and Adolescent Health Fernandes Figueira/Oswaldo Cruz Foundation under CAAE number 24734719.7.0000.5269.

The variables studied were maternal data (maternal age and ethnicity), birth data (type of delivery, Apgar score, gender, birth weight, need of cardiopulmonary resuscitation, gestational age at delivery, previous diagnosis of DS, presence of CHD, and extra-cardiac malformations), hospitalization data (NICU admission, main route of feeding, use of nutritional therapy, hypoglycemia, hypothermia, transient tachypnea of the newborn, and NICU intercurrences), and outcomes (discharge, transfer, or death, length of hospital stay, and discharge weight). Hypoglycemia was defined as blood glucose<40 mg/dL in the presence of symptoms such as tachypnea, hypothermia, tachycardia, nervousness, hypotonia, or lethargy. Hypothermia was defined as a body temperature<36.0°C. Transient tachypnea in the newborn was defined as a respiratory rate greater than 60 breaths per minute for more than 4 h.

Data were tabulated in the Epi Info program (Centers for Disease Control and Prevention, Atlanta, GA, USA) and analyzed descriptively. Categorical variables were described using absolute and percentage frequencies. Numerical variables were described by median, minimum, and maximum values. The multivariate two-step cluster method was used to classify newborns with similar profiles in terms of birth characteristics, NICU intercurrences, and outcomes. This technique makes it possible to work with categorical and numerical variables simultaneously. The number of clusters was based on the silhouette coefficient. The latter assesses the cohesion and discrimination of the groups formed and ranges from −1 to +1, where positive values greater than 0.5 indicate a reasonable partitioning of the data and values less than 0.2 indicate that the data do not exhibit a cluster structure.

The clustering method is a multivariate statistical analysis approach. This technique makes it possible to identify groups with homogeneous characteristics, which can be used when there are at least three variables. One of the most commonly used clustering techniques is k-means, which consists of dividing a set of objects into smaller subsets according to their characteristics (variables). After mathematical distance calculations, it is possible to assign a measure of proximity (similarity) to all pairs of objects and between each object and the subsets. Subsequently, in an interactive process, i.e., by repeating the previous steps, the subgroups are formed in such a way that the distances between the members of a subgroup are minimal and the distances between the subgroups are maximal^11^.

## RESULTS

A total of 119 newborns with DS were initially identified. After applying the exclusion criteria, the final sample consisted of 112 newborns. The maternal age group with the highest number of DS births was>35 years [80 cases of 112, 95% confidence interval (CI) 62.7–80.1]. The lowest maternal age was 16 years, and the highest was 42 years. The predominant maternal ethnicity was white (54.46%), followed by mixed (41.07%). DS was diagnosed during the prenatal period only in 20.53% of cases by invasive procedures, being 17.85% by amniocentesis, 1.79% by cordocentesis, and 0.89% by chorionic villus sampling.

The most common type of delivery was cesarean section (94 of 112 cases, 95%CI 75.79–90.19), and males had a slightly higher number of cases of DS (60 of 112 cases). The gestational age at delivery was distributed as follows: 53.57% term (37–42 weeks), 44.64% preterm (from 29 to 36+6 weeks), and 1.79% extremely preterm (<28 weeks). Cardiopulmonary resuscitation was required in 11.61% of newborns, and the mean birth weight was 2,580 g.

The main reason for NICU admission during the study was CHD in 57.66% of the cases studied (53 out of 112), being 30.63% of AVSD, 16.22% of ventricular septal defect, 5.41% of atrial septal defect, 3.60% of tetralogy of Fallot, and 1.80% of ventricular and atrial septal defects. The extra-cardiac malformations leading to NICU admission were duodenal atresia [9.82% (95%CI 4.94–14.7)], followed by esophageal atresia [3.57% (95%CI 0.98–8.89%)]. Table 1 shows all congenital malformations observed in DS newborns.

**Table 1:** Distribution of congenital malformations in newborns with Down syndrome.

Congenital malformations	Frequency	
	n	%
Congenital heart disease	53	57.66
Duodenal atresia	11	9.82
Esophageal atresia	4	3.57
Pyelectasis	4	3.57
Clubfeet	3	2.68
Anal imperforation	3	2.68
Hydrocele	3	2.68
Hepatomegaly	3	2.68
Hydronephrosis	2	1.79
Cryptorchidism	2	1.79
Single umbilical artery	2	1.79
Tracheoesophageal fistula	1	0.89
Hypospadias	1	0.89
Pulmonary hypoplasia	1	0.89
Inguinal hernia	1	0.89
Syndactyly	1	0.89
Choroid plexus cyst	1	0.89
Hemivertebra	1	0.89

The most common intercurrence during NICU hospitalization was transient tachypnea of the newborn, which occurred in 63.39% of cases, followed by hypoglycemia with 18.75%. During the NICU hospitalizations, 64.29% of newborns received some type of nutritional therapy, including 56.25% with enteral probing, 7.14% with cup, 11.61% with parenteral nutrition, and 0.89% with translactation (95%CI 75.79–90.19%). The NICU intercurrences in DS newborns are shown in Table 2.

**Table 2:** Neonatal intensive care unit intercurrences in newborns with Down syndrome.

Intercurrence	Frequency	
	n	%
Transient tachypnea of the newborn	71	63.39
Hypoglycemia	21	18.75
Pulmonary hypertension	8	7.14
Sepsis	8	7.14
Orotracheal intubation	7	6.25
Gastrostomy	7	6.25
Hypothermia	5	4.46
Necrotizing enterocolitis	6	5.36
Urinary tract infection	6	5.36
Metabolic acidosis	3	2.68
Colostomy	3	2.68
Seizures	1	0.89
Cardiorespiratory arrest	1	0.89
Pulmonary atelectasis	1	0.89

The mean NICU length of stay was 27 days, and the most common outcome was discharge [82.14% 95%CI (73.78–88.14)]. Notably, 12.50% of newborns were transferred to an external NICU, and only 6% died. Causes of death included sepsis (4 out of 6 cases, with only 1 being an isolated cause) and metabolic acidosis (2 out of 6 cases, both associated with PH). The other death cases were multiple congenital malformations associated with CHD and extreme prematurity. Of the six recorded deaths, four were male and two were female.

Cluster 01 (n=28) had 75% male newborns, the highest percentage of DS diagnoses in the prenatal period (35.7%), and the highest number of preterm newborns (64.3%). Cluster 01 also had the highest number of NICU intercurrences, the highest rate of orotracheal intubation and necrotizing enterocolitis, and the longest NIUC length of stay (55 days). Cluster 01 had a number of newborns with CHD similar to other groups and was the second group with the highest number of deaths.

Cluster 02 (n=11) was almost evenly split in terms of gender (45.5% female and 54.5% male) and had the highest number of cardiorespiratory resuscitations, the highest need for parenteral nutrition (36.4%), the highest number of CHD (81.8%), and no deaths.

Cluster 03 (n=40), all being female, had a higher percentage of stillbirths (70%), a higher rate of hypoglycemia during NICU hospitalization (22.5%), and a higher rate of transient tachypnea in the newborn (35.0%).

Cluster 04 (n=33), all being male, had the highest number of congenital malformations (66.7%), the highest number of deaths (9.1%), 6.1% of cases of metabolic acidosis, the lowest rate of CHD (9.4%), and the shortest NICU length of stay (12 days). Table 3 shows the variables stratified according to cluster analysis.

**Table 3:** Variables stratified according to cluster analysis.

Variables		Clusters			
		01 (n=28)	02 (n=11)	03 (n=40)	04 (n=33)
Gender					
	Female	25.0% (7)	45.5% (5)	100% (40)	0% (0)
	Male	75.0% (21)	54.5% (6)	0% (0)	100% (33)
Prenatal diagnosis of Down syndrome		35.7% (10)	27.3% (3)	25% (10)	24% (8)
	Yes				
Need for cardiopulmonary resuscitation					
	Yes	3.6% (1)	27.3% (3)	5% (2)	21.2% (7)
Gestational age					
	Preterm^1^	39.9% (19)	63.6% (7)	30% (12)	22.4% (14)
	Term^2^	32.1% (1)	36.4% (4)	70% (28)	57.6% (19)
Presence of congenital malformation					
	Yes	10.7% (3)	0% (0)	25% (10)	66.7% (22)
Nutrition					
	Formula	75% (21)	54.5% (6)	92.5% (37)	90.9% (30)
	Breastfeeding	10.7% (3)	9.1% (1)	0% (0)	3% (1)
	Parenteral	14.3% (4)	36.4% (4)	7.5% (3)	6.1% (2)
Nutritional therapy					
	Translactation	0% (0)	0% (0)	0% (0)	3% (1)
	Cup	0% (0)	0% (0)	12% (4)	12.1% (8)
	Enteral probing	67.9% (19)	54.5% (6)	55% (22)	48.5% (16)
	None	32.1% (9)	45.5% (5)	35% (14)	36.4% (12)
Hypothermia^3^					
	Sim	7.1% (2)	0% (0)	2.5% (1)	6% (2)
Hypoglicemia^4^					
	Sim	17.9% (5)	9.1% (1)	22.5% (9)	18.2% (6)
Transit tachypnea of the newborn^5^		85.7% (24)	63.6% (7)	55% (22)	54.5% (18)
	Yes				
Congenital heart disease					
	Yes	67.9% (19)	81.8% (9)	67.5% (27)	28.1% (10)
Respiratory distress					
	Yes	14.3% (4)	9.1% (1)	35% (14)	25% (28)
NICU intercurrence					
	Yes	60.7% (17)	27.3% (3)	17.5% (7)	27.3% (9)
Duodenal atresia					
	Yes	0% (0)	100% (11)	0% (0)	0% (0)
Sepsis					
	Yes	28.6% (8)	0% (0)	0% (0)	0% (0)
Orotracheal intubation					
	Sim	14.3% (4)	0% (0)	2.5% (1)	6.1% (2)
Hypoactivity					
	Yes	0% (0)	0% (0)	5% (2)	3% (1)
Necrotizing enterocolitis					
	Yes	10.7% (3)	9.1% (1)	2.5% (1)	3% (1)
Cardiorespiratory arrest					
	Yes	0% (0)	0% (0)	0% (0)	3% (1)
Metabolic acidosis					
	Yes	3.6% (1)	0% (0)	0% (0)	6.1% (2)
Pulmonary hypertension					
	Yes	10.7% (3)	9.1% (1)	7.5% (3)	3% (1)
Outcome					
	Discharge	67.9% (19)	81.8% (9)	87.5% (35)	87.9% (29)
	Transferring	25% (7)	18.2% (2)	10% (4)	3% (1)
	Death	7.1% (2)	0% (0)	2.5% (1)	9.1% (3)

1 Premature: <36+6 weeks;

2 Term>37 weeks;

3 Body temperature<36.0°C;

4 Capillary blood glucose<40 mg/dL;

5 Respiratory rate>60 incursions per minute.

NICU: neonatal intensive care unit. Values in relative (%) and absolute frequency.

## DISCUSSION

In this study, we evaluated the incidence of associated congenital malformations, NICU intercurrences, and outcomes of newborns diagnosed with DS in three NICUs in the city of Rio de Janeiro, Brazil. According to Moreira and Gusmão^12^, DS occurs as a free trisomy (not a chromosomal disjunction) in 95% of cases. In this study, 98.2% of the cases had this type of trisomy, while 1.78% had a translocation. There was no difference in severity between the newborns who had a translocation in the karyotype.

Screening and prenatal diagnosis programs for the diagnosis of DS in different populations have proven to be very important so that these fetuses can be referred to referral health units in a timely manner^13,14^. In this study, 27.68% of the newborns were diagnosed with DS before birth.

Congenital heart disease was the most common congenital malformation in DS newborns, with a rate of 57.66% of our cases, with AVSD being the most prevalent (30.66%). These findings are in agreement with Fudge et al.^15^ who retrospectively analyzed 4,350 patients with DS and observed that AVSD was the most common CHD. Taura et al.^16^ assessed the prevalence of CHD among 42 patients with DS in southwestern Saudi Arabia. They observed a prevalence of 81% of CHD, with atrial septal defect (28.5%) being the most common, followed by ventricular septal defect (25%), patent ductus arteriosus (16%), and AVSD (14.3%).

Compared to other congenital malformations in newborns with DS, we observed a higher incidence of duodenal atresia (9.82%), followed by esophageal atresia (3.57%). Bermudez et al.^6^ retrospectively reviewed data from 1,207 patients with DS and observed a rate of 5% of gastrointestinal malformations, including 13 cases of duodenal atresia, 8 of imperforate anus, 4 annular pancreases, 2 congenital megacolon, 2 esophageal atresias, 2 esophageal compression by an anomalous subclavian, and 1 case of duodenal membrane. Buchin et al.^17^ retrospectively reviewed data from 187 patients with DS, and gastrointestinal malformations were observed in 27 (14.4%), similar to our results.

The most common NICU intercurrence in DS newborns was the transient tachypnea of the newborn. MacAndrew et al.^18^ in a retrospective cohort analyzed full-term infants with (4,623) and without (606,770) DS. Infants with DS had more respiratory distress, thrombocytopenia, feeding problems, and PH. They received respiratory support for a longer period of time and had a longer length of stay. Infants with DS have a high risk of oral motor difficulties and pharyngeal dysphagia with aspiration, both of which require systematic attention. Clinical interventions should promote swallowing safety and the development of feeding abilities^19^. In our study, 64.29% of DS newborns received some type of nutritional therapy, including 56.25% with enteral probing.

Cluster 02 included all cases of duodenal atresia, which is directly related to the higher number of parenteral nutrition cases (36.4%). In terms of gender, this cluster had the smallest difference between female and male cases (45.5 and 54.5%, respectively). The higher number of premature newborns found in this group may be related to the finding of a greater need for cardiopulmonary resuscitation at birth.

Clusters 03 and 04 differed significantly in terms of gender, but the most striking differences were the higher number of full-term births, cases of CHD, and respiratory distress in females. The cluster formed by males, on the contrary, had a higher number of cases of prematurity, congenital malformations, cardiorespiratory arrest during NICU hospitalization, and metabolic acidosis. No data on significant gender differences in morbidity or mortality in DS were found in the literature.

Congenital heart disease was present in a higher proportion in cluster 02, which was not reflected in the length of hospital stay or the outcome. Therefore, we can assume that the outcome of death was not directly related to CHD, since the group with the highest number of this outcome was the cluster with the lowest number of newborns with CHD. This suggests that CHD was diagnosed and corrected early and that surgical correction can be considered a protective factor against death.

## CONCLUSION

Down syndrome newborns are at higher risk of NICU admission and longer hospital stays due to common congenital abnormalities and prematurity. There is a need for further studies targeting those born with DS in order to improve the quality of neonatal care for them.
